# Developing the first national database and map of lymphatic filariasis clinical cases in Bangladesh: Another step closer to the elimination goals

**DOI:** 10.1371/journal.pntd.0007542

**Published:** 2019-07-15

**Authors:** Mohammad J. Karim, Rouseli Haq, Hayley E. Mableson, A. S. M. Sultan Mahmood, Mujibur Rahman, Salim M. Chowdhury, A. K. M. Fazlur Rahman, Israt Hafiz, Hannah Betts, Charles Mackenzie, Mark J. Taylor, Louise A. Kelly-Hope

**Affiliations:** 1 Filariasis Elimination and STH Control Programme, Communicable Disease Control, Directorate General of Health Services, Ministry of Health and Family Welfare, Dhaka, Bangladesh; 2 Centre for Neglected Tropical Diseases, Department of Tropical Disease Biology, Liverpool School of Tropical Medicine, Liverpool, United Kingdom; 3 Centre for Injury Prevention and Research, Dhaka, Bangladesh; NIH-TRC-ICER, INDIA

## Abstract

**Background:**

The Bangladesh Lymphatic Filariasis (LF) Elimination Programme has made significant progress in interrupting transmission through mass drug administration (MDA) and has now focussed its efforts on scaling up managing morbidity and preventing disability (MMDP) activities to deliver the minimum package of care to people affected by LF clinical conditions. This paper highlights the Bangladesh LF Programme’s success in conducting a large-scale cross-sectional survey to determine the number of people affected by lymphoedema and hydrocoele, which enabled clinical risk maps to be developed for targeted interventions across the 34 endemic districts (19 high endemic; 15 low endemic).

**Methodology/Principal findings:**

In the 19 high endemic districts, 8,145 community clinic staff were trained to identify and report patients in their catchment area. In the 15 low endemic districts, a team of 10 trained field assistants conducted active case finding with cases reported via a SMS mHealth tool. Disease burden and prevalence maps were developed, with morbidity hotspots identified at sub-district level based on a combination of the highest prevalence rates per 100,000 and case-density rates per square kilometre (km^2^). The relationship between morbidity and baseline microfilaria (mf) prevalence was also examined. In total 43,678 cases were identified in the 19 high endemic districts; 30,616 limb lymphoedema (70.1%; female 55.3%), 12,824 hydrocoele (29.4%), and 238 breast/female genital swelling (0.5%). Rangpur Division reported the highest cases numbers and prevalence of lymphoedema (26,781 cases, 195 per 100,000) and hydrocoele (11661 cases, 169.6 per 100,000), with lymphoedema predominately affecting females (n = 21,652). Rangpur and Lalmonirhat Districts reported the highest case numbers (n = 11,199), and prevalence (569 per 100,000) respectively, with five overlapping lymphoedema and hydrocoele sub-district hotspots. In the 15 low endemic districts, 732 cases were identified; 661 lymphoedema (90.2%; female 39.6%), 56 hydrocoele (7.8%), and 15 both conditions (2.0%). Spearman’s correlation analysis found morbidity and mf prevalence significantly positively correlated (r = 0.904; p<0.01).

**Conclusions/Significance:**

The Bangladesh LF Programme has developed one of the largest, most comprehensive country databases on LF clinical conditions in the world. It provides an essential database for health workers to identify local morbidity hotspots, deliver the minimum package of care, and address the dossier elimination requirements.

## Author summary

The Global Programme to Eliminate Lymphatic Filariasis (GPELF) requires lymphatic filariasis (LF) endemic countries, such as Bangladesh, to estimate the number of lymphoedema and hydrocoele cases in order to deliver the minimum package of care required to control morbidity and reduce patient suffering. This paper highlights the Bangladesh LF Elimination Programme’s progress in training more than 8000 community health workers to identify more than 44,000 cases across 34 endemic districts where approximately 70 million people are at risk. The morbidity data collected enabled the creation of a national database and a series of risk maps of lymphoedema and hydrocoele to be developed, which highlighted the significant burden in northern Rangpur Division, especially of lymphoedema among female patients. The Bangladesh LF Elimination Programme’s efforts to identify LF cases across all endemic districts represents one of the most comprehensive national databases on LF clinical cases in the world. It provides an informative database for health workers to use in the delivery of the minimum package of care and a template for other countries to adopt and develop national strategies to manage morbidity and prevent disability as recommended by GPELF.

## Introduction

The Bangladesh Lymphatic Filariasis (LF) Elimination Programme has made great strides towards eliminating LF as a public health problem [1]. The LF Programme was initiated in 2001 following the launch of the World Health Organization’s (WHO) Global Programme to Elimination Lymphatic Filariasis (GPELF) [2]. It began the elimination process by addressing the GPELF’s first aim of interrupting transmission through mass drug administration (MDA) [1,3,4]. At the start of the Bangladesh LF Programme, an estimated 70 million people were at risk of LF infection; caused by the parasite *Wuchereria bancrofti* and transmitted by the *Culex* sp. mosquitoes. Furthermore, tens of thousands of people were estimated to suffer from LF clinical manifestations, including lymphoedema and hydrocoele [5–8].

In Bangladesh in 2001, LF was endemic in 34 of the 64 districts. Of the 34 endemic districts, 19 districts were eligible for MDA based on the baseline antigenaemia (Ag) and microfilaria (mf) prevalence rates between 1% and 19%, and known historical presence of patients [1,4–8]. Over the past two decades MDA has been successfully scaled up across the 19 endemic areas with the interruption of transmission confirmed through transmission assessment surveys (TAS) [1]. The other 15 endemic districts were classified as low-endemic not requiring MDA as mf prevalence rates were below 1% [9]. Their low endemicity has also been confirmed through TAS [1], and recent molecular xenomonitoring for *W*. *bancrofti* in *Culex quinquefasciatus* in two districts in the northern endemic region [10]. Collectively, these implementation activities and impact assessments provide evidence that the Bangladesh programme is on target to meet the GPELF’s first aim of interrupting transmission [2].

However, in order for the Bangladesh LF Programme to meet all of the GPELF’s elimination requirements, it needed to address the second aim of alleviating suffering of patients by managing morbidity and preventing disability (MMDP) [2,11,12]. This component requires that a country must be able to document: (i) the number of patients with lymphoedema and hydrocoele (reported or estimated) by implementation unit (IU); (ii) 100% geographical coverage of the availability of the recommended minimum package of care; and (ii) the readiness and quality of available services, in selected designated facilities [13]. The estimated number of cases per IU allows for the proper planning and provision of services, namely the minimum package of care which comprises: (i) treatment of episodes of acute dermatolymphangiodenitis (ADLA); (ii) preventing ADLA and progression of lymphoedema to elephantiasis; (iii) ensuring access to hydrocoele surgery; and (iv) providing anti-filarial medicines to treat infection [11].

The prevention of disability due to LF clinical conditions is already a major integrated component of the Bangladesh LF Programme with the following objectives:

- To list the community clinics in filariasis endemic areas for providing care
- To find and report filariasis patients in endemic districts
- To train caregivers at community clinics
- To arrange regular management sessions at community clinics
- To train patients and family caregivers on management at community clinic and at home

Further, the revitalisation of community clinics is one of the priorities of the Bangladesh Government’s health sector. Community clinics are small clinics at the grass root level and their main purpose is to provide first level primary care, including health education, health promotion, first aid and treatment of minor ailments to a catchment population of 6,000 to 10,000. The LF Programme aimed to maximise the use of this existing health infrastructure, and train community clinic staff to identify and report cases, as well as provide health education and information on home-based long-term care to patients and their families and caregivers. This will help to ensure that patients who are not fully capable of self-care can maintain the highest possible quality of life, degree of independence, personal fulfilment and dignity. It will also help to identify high prevalence areas, optimise the distribution of resources and determine where additional surveillance may be required to show impact.

The aim of this paper was to describe the way in which the Bangladesh LF Programme worked to provide MMDP training to upazila and community clinic staff across all endemic areas to enable them to search, identify and report the number of clinical cases found in their community clinic catchment area. Further, the number of clinical cases, prevalence and case density rates were quantified and mapped to determine the distribution of lymphoedema and hydrocoele, as well as to help identify geographical hotspots, and determine if there was a relationship with known high-risk transmission areas so that post-elimination patient care and surveillance may be optimised.

## Methods

### Survey sites

**Fig 1:**
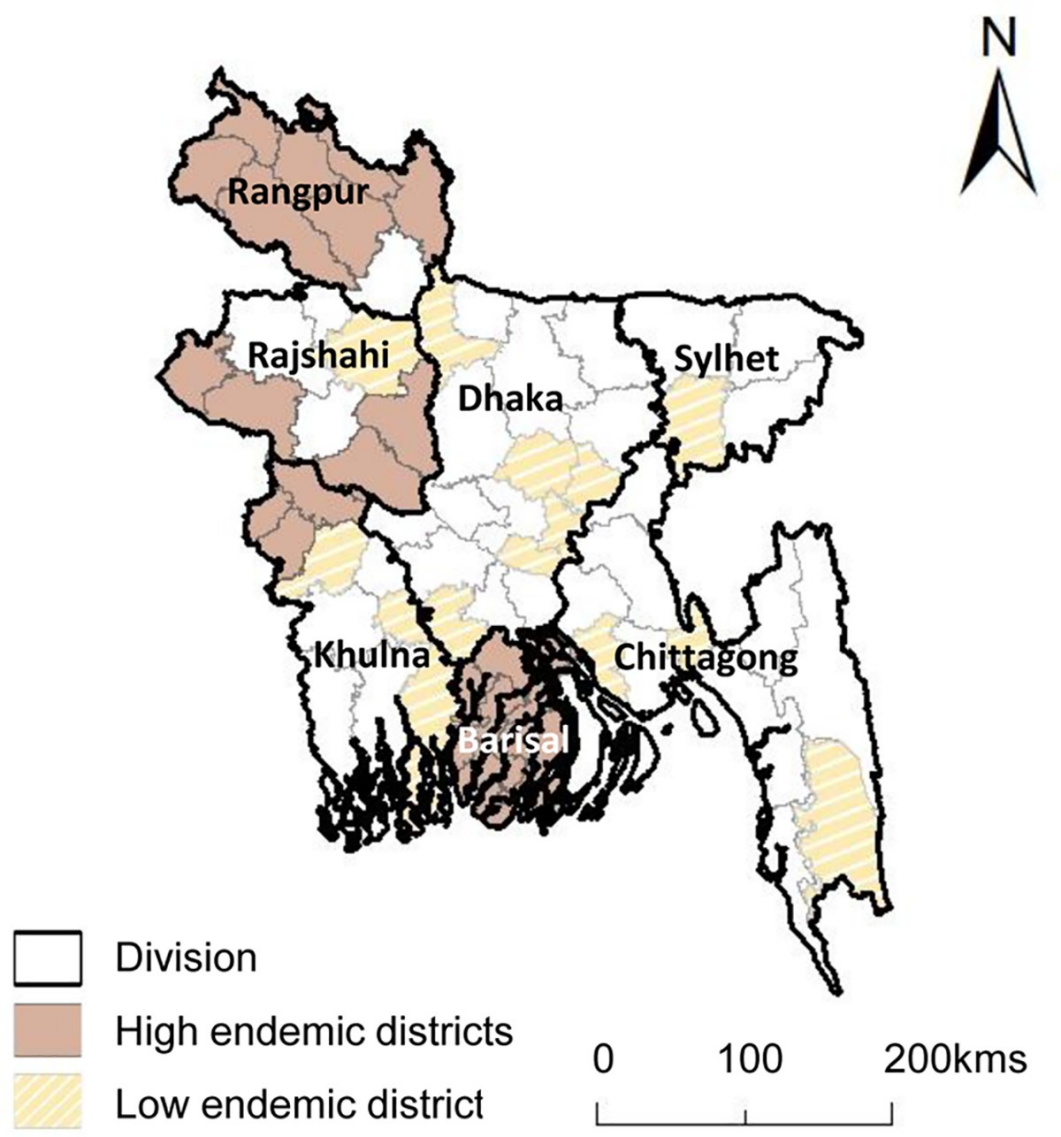
Bangladesh high and low endemic districts by division.

The 19 high endemic districts (that received MDA) considered to be the most endemic are in five of the eight administrative divisions of Bangladesh, which broadly group into three regions as shown in Fig 1. In the northern region, districts include Dinajpur, Kurigram, Lalmonirhat, Nilphamari, Panchagarh, Rangpur and Thakurgaon. In the central western region, the districts include Chuadanga, Kushtia, Meherpur, Chapainawabganj, Pabna, Rajshahi and Sirajganj. In the southern region, the districts include Barguna, Barisal, Jhalokathi, Patuakhali and Pirojpur.

The 15 low endemic districts (that did not require MDA) and considered to be the least endemic, are in five administrative divisions spread across the country, and include Bagerhat, Bandarban, Bogra, Feni, Gazipur, Gopalganj, Habiganj, Jamalpur, Jhenaidaha, Laxmipur, Munshiganj, Narayanganj, Narail, and Narshingdi, and peri-urban areas of Dhaka. Details of all districts baseline mf rates, year of patient searching, and population estimates are summarised in Table 1.

**Table 1:** District baseline prevalence of infection, year of training and patient searching, and population estimates.

Districts	Endemicity	Baseline Mf (%)	Year of training and patient searching	Population		
				Male	Female	Total
Rangpur Division						
Dinajpur	High	4.8	2013	1,545,340	1,517,466	3,062,806
Kurigram	High	6.0	2013	1,035,002	1,084,567	2,119,569
Lalmonirhat	High	16.0	2013	644,083	642,547	1,286,630
Nilphamari	High	10.0	2013	945,397	933,416	1,878,814
Panchagarh	High	10.8	2013	508,798	502,851	1,011,650
Rangpur	High	10.0	2013	1,478,909	1,472,204	2,951,113
Thakurgaon	High	16.0	2013	718,326	705,502	1,423,828
Barisal Division						
Barguna	High	2.6	2015	458,989	477,830	936,819
Barisal	High	1.0	2016	1,207,626	198,684	2,468,229
Jhalokathi	High	1.0	2016	349,527	375,412	724,939
Patuakhali	High	2.0	2015	790,606	821,007	1,630,501
Pirojpur	High	2.2	2016	582,174	600,015	1,182,189
Khulna Division						
Bagherhat	Low	0.0	2016	785,967	781,521	1,567,488
Chuadanga	High	0.8	2015	592,680	592,026	1,184,706
Jhenaidaha	Low	0.0	2017	941,287	939,694	1,880,981
Kushtia	High	0.2	2015	1,021,539	1,021,331	2,042,870
Meherpur	High	1.0	2015	340,647	347,073	687,721
Narail	Low	0.0	2016	375,417	390,936	766,353
Rajshahi Division						
Bogra	Low	0.0	2016	1,814,613	1,796,839	3,611,453
Chapainawabganj	High	8.4	2014	839,986	868,066	1,708,053
Pabna	High	2.4	2014	1,309,335	1,306,548	2,615,883
Rajshahi	High	1.0	2014	1,358,017	1,332,530	2,690,547
Sirajganj	High	1.2	2014	1,608,367	1,602,927	3,211,294
Dhaka Division						
Gazipur	Low	0.0	2016	1,885,235	1,729,443	3,614,679
Gopalgonj	Low	0.0	2017	613,649	631,361	1,245,010
Jamalpur	Low	0.0	2017	1,198,613	1,236,021	2,434,634
Munshiganj	Low	0.0	2016	766,230	768,944	1,535,174
Narayanganj	Low	0.0	2016	1,615,644	1,515,124	3,130,768
Narshingdi	Low	0.0	2016	1,171,236	1,191,474	2,362,710
Peri-urban Dhaka	Low	0.05	2016	1,157,348	1,177,346	2,334,694
Chittagong Division						
Bandardon	Low	0.0	2016	215,941	196,439	412,380
Feni	Low	0.0	2016	737,108	789,264	1,526,372
Laxmipur	Low	0.0	2016	879,035	957,222	1,836,258
Sylhet Division						
Hobiganj	Low	0.0	2016	1,089,095	1,129,255	2,218,350
						
** Total**	-	-	-	**32,581,766**	**31,632,885**	**65,295,465**

### Health worker training

For the 19 high endemic districts, training was conducted in phases between 2013 and 2016. Training materials were developed by Bangladesh LF Programme using the standard WHO guidelines [11]. All staff attended a one-day training session which included background on LF in Bangladesh, MMDP, and identification and reporting of clinical cases of lymphoedema and hydrocoele. A cascade model of training was used, which included the delivery of training to community clinic staff though layers of trainers. First, the central LF Programme master trainer group conducted advocacy and training for the district trainer group, which comprised of civil surgeons, the Divisional Director of Family Planning, the Upazila (sub-district) Health and Family Planning Officer, Medical Officer, and the Medical Officer of Disease Control/Resident Medical Officer. The district trainer group then conducted advocacy and training for the Upazila (sub-district) trainer group, which comprised the Upazila Health and Family Planning Officer, Medical Officer of Disease Control, and the Upazila Health Inspectors. The upazila trainer group then conducted training for the community clinic staff, which comprised Community Health Care Providers, Health Assistants, and Family Welfare Advisors, and who were the data collectors responsible for searching and reporting patients found in their clinic catchment area.

### Patient searching in high endemic districts

The cross-sectional patient searching activity was conducted over a 10-day period in phases between 2013 and 2016 following the training of community clinic staff. Community clinic staff were responsible for collecting the data by visiting each household in their respective clinic catchment area, visiting around 40 households per day. To verify that all households had been visited, community clinic data collectors were required to record contact information of 20 households visited each day, regardless of the presence of patients, which would be verified by the supervisors in that area.

Data collectors recorded information for all identified patients on a paper form. Data recorded at community level included demographic information (district, upazila, union, ward, community clinic name, patient name, family head name, patient address, age, gender), condition information of lymphoedema (right/left arm, right/left leg, right/left breast), and hydrocoele. Information on ADLAs were not collected systematically and therefore not analysed.

A summary of the data was sent to the LF Programme at central level and included district, upazila, lymphoedema (arm, leg, breast) by sex, and hydrocoele, which was used in this study. No complete district-level age-specific data was available, however selected upazilas had detailed age-specific data for each condition and lymphoedema stage data, which were used to examine the number and proportion of cases across different age groups and different lymphoedema stages. The stage of lymphoedema was recorded according to the Dreyer method [14], and grouped into mild (stages 1–2), moderate (stages 3–4; enlarged limb with shallow folds), and severe (stages 5–7; greatly enlarged with deep folds).

### Patient searching in low endemic districts

The cross-sectional patient searching activity was conducted in the peri-urban areas of Dhaka district in 2015, utilising the same methods as the high endemic districts where the community clinic staff were trained and conducted the patient searching activities in their catchment areas. Data collection was conducted over a 10-day period using paper forms as in the endemic districts. Summary data for each case was also reported using the mHealth system *MeasureSMS-morbidity* [15–19] and cross-checked against the paper forms to ensure no duplication. Health workers reported a summary of each case identified via a mobile phone short message service (SMS) text, reporting the location (upazila, union), age, sex, condition (lymphoedema, hydrocoele or both conditions), the severity (for lymphoedema) and number of acute attacks in the last six months.

In the remaining 14 low endemic districts, data collection was conducted between February 2016 and January 2017 using paper forms as in the endemic districts, and summary data was reported through the *MeasureSMS* system. Data collection was carried out by a centrally-trained mHealth team of 10 data collectors that would visit each of the districts and conduct patient searching by visiting the upazila health facilities and gathering records of any known patients, and then would conduct house-to-house searching for patients in each upazila, confirming health facility records, and recording any additional patients identified through community informants. Data collection was conducted on average for a 10-day period, and all patients were reported by SMS to the *MeasureSMS* system.

### Data analysis, mapping and hotspot identification

For the high endemic districts, all data were collected by paper, then entered into Microsoft Excel Version 12.3.6 (Microsoft Corp., Redmond, VA, USA) and analysed using IBM SPSS Statistics 32 (IBM Corp., Armonk, NY, USA). Data were tabulated to determine the total number of lymphoedema, hydrocoele and other conditions (namely breast and female genital lymphoedema) at district and upazila level. For the low endemic districts, data were downloaded from the *MeasureSMS* database into Microsoft Excel, with the count of each condition tabulated at district level. A summary of the different patient searching and reporting methods and associated costs are presented in Table 2.

**Table 2:** Summary of data collection methods and costs in different endemic settings.

Group	Data collection methods	Data collectors and of average days searching	Data reporting mechanism	Mean cost per district Bangladeshi Taka BDT (US$ estimated cost*)
High endemic districts (n = 19)	Census	1203 health assistants 10 days	Paper forms	425,000 BDT (US$ 12,952)
Low endemic peri-urban district (n = 1)	Census	87 health assistants 10 days	Paper forms and mHealth (MeasureSMS)	657,493 BDT (US$ 7,922)
Low endemic districts (n = 14)	Case finding via health facility data	10 data collectors 10 days each district	mHealth (MeasureSMS)	245,657 BDT (US$ 7,177)

*US$ exchange based on April 2019 rates

For all districts, clinical prevalence estimates per 100,000 people were calculated using the population estimates based on the year of patient searching, which were extrapolated from the 2011 census using the annual growth rates [20]. District populations are summarised in Table 1. For high endemic districts, prevalence rates at upazila level were also calculated to better help to identify focal areas of high clinical prevalence. In addition, to account for the different geographical sizes of the upazilas and to better understand the spatial density of lymphoedema and hydrocoele cases, the case-density per square kilometre (km^2^) was calculated.

The geographic distributions of lymphoedema and hydrocoele cases, prevalence rates and case- density rates were mapped at the district and upazila level. Maps were created in in ArcGIS 10.5.1 (ESRI, Redlands, California) using administrative boundaries available from GeoDASH (https://geodash.gov.bd). To identify potential morbidity hotspots i.e. high risk areas that could be readily identified by LF Programme to prioritise MMDP interventions, the 10 upazilas with the highest case prevalence rates per 100,000 and highest case-density rates per km^2^ were selected and mapped together, to identify the upazilas that had both the highest prevalence and case-density rates.

Further, to account for the impact of previous hydrocoele surgery campaigns and routine hospital surgeries conducted in the high endemic districts between 2003 and 2012 [21], the number of hydrocoele surgeries recorded from the districts were summarised from annual reports.

### Relationship between district baseline mf prevalence and clinical prevalence rates

To determine if there is a relationship between the baseline mf prevalence, and the clinical case prevalence rates, the district baseline mf prevalence rates recorded at the start of the programme before the upscaling of MDA were correlated with the clinical prevalence rates of all total cases, lymphoedema, and hydrocoele) using Spearman’s correlation.

Further, simple linear regressions using the *ln* function of the R statistical package (R Core Team, 2013) were used to determine if mf prevalence could predict the overall prevalence, lymphoedema prevalence and hydrocoele prevalence.

### Ethics statement

The LF patient identification and reporting activities are part of routine programme activities conducted by the Ministry of Health and Family Welfare (MOHFW), Bangladesh, and therefore ethical clearance and written consent in Bangladesh was not required. Patients were informed of the procedures, and oral consent was given to record and report the details relating to their conditions, however this was not documented. Ethical approval was obtained from the Liverpool School of Tropical Medicine Research Ethics Committee (Research Protocol 12.22) to support MOHFW programme activities and analysis of data. All data were analysed anonymously.

## Results

### District-level training, case numbers and prevalence rates

#### High endemic districts

Health worker training and patient searching activities were conducted between 2013 and 2016 across the 19 high endemic districts, with an estimated total population of 34.8 million. A total of 3,396 community clinics (mean 179; range 75–313 per district) were provided with materials to deliver the minimum package of care at the community level (Table 3). A total of 1,313 upazila level Health Inspectors and Assistant Health Inspectors (HI/AHI) (mean 61; range 21–119 per district), and 8,145 community clinic staff (mean 429; range 62–939 per district) were trained in MMDP practices, and to identify and report cases of lymphoedema and hydrocoele in their community, regardless of causality, to help ensure all patients would receive care. The average district cost of this activity was 1,074,982 BDT (est. US$12,952) (Table 2).

**Table 3:** Number of community clinics per high endemic district, and number of upazila level Health Inspectors/Assistant Health Inspectors (HI/AHI), and community clinic staff trained.

District	Number of Community Clinics	HI/AHI	Community clinic staff*
Rangpur Division			
Dinajpur	313	119	939
Kurigram	267	86	801
Lalmonirhat	146	52	438
Nilphamari	162	71	486
Panchagar	91	53	273
Rangpur	307	96	921
Thakurgaon	136	61	408
Barisal division			
Barguna	102	48	110
Barisal	258	96	310
Jhalkathi	83	36	141
Patuakhal	180	80	156
Pirojpur	140	60	201
Khulna division			
Chuadanga	91	39	149
Kushtia	195	71	201
Meherpur	75	21	61
Rajshahi division			
Chapainawabganj	129	55	387
Pabna	230	87	690
Rajshahi	205	87	615
Sirajganj	286	95	858

Total	3396	1313	8145

* Community clinic staff include Community Health Care Providers, Health Assistants, and Family Welfare Advisors

In total, 43,678 clinical cases were identified and reported from the 19 high endemic districts, which comprised 30,616 cases of leg and/or arm lymphoedema (70.1%), 12,824 cases of hydrocoele in men (29.4%), and 238 cases of female breast lymphoedema or genital swelling (0.5%) (Table 4). Overall, the number of leg and/or arm lymphoedema cases was 3.7 times higher in females (n = 24,150) than males (n = 6,466). Regionally, the highest number of cases, including all conditions, was reported in the northern Rangpur Division (n = 38,582; district mean = 5512, 95% CIs 2489–8535), with two-thirds of cases within Rangpur Division from three districts; Rangpur (n = 11,199), Lalmonirhat (n = 7,320) and Nilphamari (n = 7,240) Districts. The number of cases in other divisions were 12 to 177 times lower than Rangpur Division, with the central Barisal Division reporting 3,129 cases (district mean = 626, 95% CIs 312–940), the southern Rajshahi Division reporting 1,749 cases (district mean = 437, 95% CIs 0–942) and Khulna Division reporting only 218 cases (district mean = 73, 95% CIs 0–272).

**Table 4:** Case numbers and prevalence rates of lymphoedema and hydrocoele by sex in 19 endemic districts.

District	Lymphoedema Cases			Lymphoedema prevalence (per 100,000)			Hydrocoele cases	Hydrocoele prevalence (per 100,000 males)	Total cases (all conditions)	Total prev (per 100,000)
	Male	Female	Total	Male	Female	Total				
Rangpur Division										
Dinajpur	356	1,688	2,044	23.0	111.2	66.7	1,021	66.1	3,067	100.1
Kurigram	162	1,077	1,239	15.7	99.3	58.5	699	67.5	1,958	92.4
Lalmonirhat	1,077	4,306	5,383	167.2	670.1	418.4	1,937	300.7	7,320	568.9
Nilphamari	820	4,655	5,475	86.7	498.7	291.4	1,715	181.4	7,240	385.3
Panchagarh	224	1,117	1,341	44.0	222.1	132.6	1,634	321.1	2,980	294.6
Rangpur	1,743	6,789	8,532	117.9	461.1	289.1	2,654	179.5	11,199	379.5
Thakurgaon	747	2,020	2,767	104.0	286.3	194.3	2,001	278.6	4,818	338.4
**Subtotal**	**5,129**	**21,652**	**26,781**	**74.6**	**315.7**	**195.0**	**11,661**	**169.6**	**38,582**	**280.9**
Barisal division										
Barguna	208	334	542	45.3	69.9	57.9	466	101.5	1,023	109.2
Barisal	124	303	427	10.3	152.5	17.3	50	4.1	478	19.4
Jhalokathi	222	370	592	63.5	98.6	81.7	49	14.0	641	88.4
Patuakhali	167	256	423	21.1	31.2	25.9	202	25.6	636	39.0
Pirojpur	121	193	314	21	32	27	31	5.3	351	29.7
**Subtotal**	**842**	**1,456**	**2,298**	**24.8**	**58.9**	**33.1**	**798**	**23.5**	**3,129**	45.1
Khulna division										
Chuadanga	23	30	53	3.9	5.1	4.5	5	0.8	59	5.0
Kushtia	74	71	145	7.2	7.0	7.1	7	0.7	159	7.8
Meherpur	0	0	0	-	-	-	0	-	0	0.0
**Subtotal**	**97**	**101**	**198**	**5.0**	**5.2**	**5.1**	**12**	**0.6**	**218**	**5.6**
Rajshahi division										
Chapainawabganj	141	552	693	16.8	63.6	40.6	201	23.9	912	53.4
Pabna	82	147	229	6.3	11.3	8.8	67	5.1	316	12.1
Rajshahi	74	135	209	5.4	10.1	7.8	26	1.9	247	9.2
Sirajganj	101	107	208	6.3	6.7	6.5	59	3.7	274	8.5
**Subtotal**	**398**	**941**	**1,339**	**7.8**	**18.4**	**13.1**	**353**	**6.9**	**1,749**	**17.1**

**Total**	**6,466**	**24,150**	**30,616**	**37.3**	**147.2**	**87.9**	**12,824**	**74.0**	**43,678**	**125.4**

The overall clinical case prevalence for the 19 high endemic districts was 125.4 per 100,000 population; for leg and/or arm lymphoedema it was 87.9 per 100,000, and for hydrocoele 74.0 per 100,000 (Table 4). Overall, the leg and/or arm lymphoedema prevalence rate was significantly higher in females (147.2 per 100,000) than males (37.3 per 100,000) (OR 3.85; 95% CIs 3.74–3.96; P< 0.0001). Regionally, the highest prevalence rate, including all conditions, was reported in Rangpur Division (280.9 per 100,000), with the highest district rates in Lalmonirhat (568.9), Nilphamari (385.3), and Rangpur (379.5). The prevalence rates in other divisions were 6 to 50 times lower than Rangpur Division, with the central Barisal Division rate 45.1 per 100,000 (district range 29.7–109.2), Rajshahi Division 17.1 per 100,000 (district range 8.5–53.4) and the lowest rate in Khulna Division 5.6 per 100,000 (district range 0–7.8).

#### Low endemic districts

Health worker training and patient searching activities were conducted between 2015 and 2017 across the 15 low endemic districts, with an estimated total population of 30.5 million. Peri-urban Dhaka was mapped first in 2015, with a total of 93 community clinic staff trained to identify and report LF clinical conditions. The remaining 14 districts were completed in 2016 and 2017 by a team of 10 data collectors. The cost of this activity in Dhaka was 657, 493 BDT (est. US$ 7,922) and the average cost of the remaining districts was 590,714 BDT (est. US$7,114) (Table 2).

In total, 732 clinical cases were identified and reported from the 15 low endemic districts, which comprised 676 cases of lymphoedema (92.3%), 71 cases of hydrocoele in men (9.7%), and 15 men found with both conditions (2.0%) (Table 5). Overall, the number of leg and/or arm lymphoedema cases was 1.6 times higher in males (n = 415) than females (n = 261). Regionally, the highest number of cases, including all conditions, was reported in the Dhaka Division across 7 districts (n = 257) and Khulna Division across three districts (n = 225), with the highest number from Narshingdi District (n = 58) and Bagherhat District (n = 136) respectively. The lowest regional number of cases was reported in Sylhet Division in one district (n = 48).

**Table 5:** Case numbers and prevalence rates of lymphoedema and hydrocoele by sex in 15 low endemic districts.

Districts	Lymphoedema cases			Lymphoedema prevalence (per 100,000)			Hydrocoele cases	Hydrocoele prevalence (per 100,000 males)	Total cases (all conditions)	Total Prevalence (per 100,000)
	Male	Female	Total	Male	Female	Total				
Dhaka division										
Gazipur	28	9	37	1.5	37	1.0	1	0.1	37	1.0
Gopalgonj	4	1	5	0.7	5	0.4	-	-	5	0.4
Jamalpur	25	4	29	2.1	31	1.3	3	0.3	31	1.3
Munshiganj	29	18	47	3.8	48	3.1	2	0.3	48	3.1
Narayanganj	23	6	29	1.4	29	0.9	2	0.1	29	0.9
Narshingdi	38	20	58	3.2	76	3.2	19	1.6	76	3.2
Peri-urban Dhaka	11	16	27	1.0	31	1.3	6	0.5	31	1.3
**Subtotal**	**158**	**74**	**232**	**1.9**	**0.9**	**1.4**	**33**	**0.4**	**257**	**1.5**
Khulna division										
Bagherhat	62	74	136	7.9	138	8.8	3	0.4	138	8.8
Jhenaidaha	23	18	41	2.4	42	2.2	1	0.1	42	2.2
Narail	23	18	41	6.1	45	5.9	4	1.1	45	5.9
**Subtotal**	**108**	**110**	**218**	**5.1**	**5.2**	**5.2**	**8**	**0.4**	**225**	**5.3**
Chittagong division										
Bandardon	6	3	9	2.8	10	2.4	1	0.5	10	2.4
Feni	36	17	53	4.9	57	3.7	7	0.9	57	3.7
Laxmipur	48	13	61	5.5	72	3.9	11	1.3	72	3.9
**Subtotal**	**90**	**33**	**123**	**4.9**	**1.7**	**3.3**	**19**	**1.0**	**139**	**3.7**
Rajshahi division										
Bogra	34	28	62	1.9	63	1.7	3	0.2	63	1.7
Sylhet division											Sylhet division
Hobiganj	25	16	41	2.3	48	2.2	8	0.7	48	2.2
	
Total	415*	261	676*	2.7	1.7	2.2	71*	0.5	732	2.4

* Includes 15 males reported with both lymphoedema and hydrocoele in ten districts (3 cases in Feni; 2 cases in Bogra, Narayanganj and Peri-urban Dhaka; 1 case in Gazipur, Jamalpur, Munshiganj, Narshingdi).

The overall clinical case prevalence for the 15 low endemic districts was 2.4 per 100,000; for leg and/or arm lymphoedema it was 2.4 per 100,000, and for hydrocoele 0.5 per 100,000 (Table 5). Overall the leg and/or arm lymphoedema prevalence was significantly higher in males (2.7 per 100,000) than females (1.7 per 100,000) (OR 1.6, 95% CIs 1.4–1.9 P< 0.0001). Regionally, the highest prevalence rate, including all conditions, was found in Khulna Division (5.3 per 100,000) and Chittagong Division (3.7 per 100,000), with the highest districts rates within those divisions found in Bagherhat District (8.8 per 100,000) and Laxmipur District (3.9 per 100,000) and respectively. The lowest regional prevalence rate was found in Dhaka Division (1.5 per 100,000).

### Upazila-level case numbers, prevalence rates, case-density rates

In total, there were 132 upazilas in the 19 high endemic districts. Overall, the mean number of cases, prevalence and density rates per upazila were 331 cases (95% CIs 233–429), 123.3 per 100,000 (95% CIs 89.6–157.1) and 1.3 cases per km^2^ (95% CIs 0.9–1.6) respectively as shown in Table 6. The highest mean number of cases, clinical prevalence and case density rates were found in Rangpur Division, which were 757 (95% CIs 552–962), 271.6 (95% CIs 202.7–340.5) and 2.8 (95% CIs 2.0–3.6) respectively, and significantly higher than the other Divisions.

**Table 6:** Mean upazila level case numbers, clinical prevalence rates per 100,000 and case-density rates per km^2^ for the high endemic divisions.

Division	No. of upazilas	Case numbers	Clinical prevalence per 100,000	Case density per km2
**Rangpur (n = 51)**	All cases	757 (552–962)	271.6 (202.7–340.5)	2.8 (2.0–3.6)
	Lymphoedema	525 (370–681)	182.8 (134.3–231.3)	2.05 (1.3–2.5)
	Hydrocoele	229 (164–294)	87.9 (60.8–115.1)	0.9 (0.5–1.1)
**Barisal** **(n = 32)**	All cases	98 (56–139)	56.9 (30.3–83.4)	0.5 (0.3–0.7)
	Lymphoedema	72 (43–101)	42.3 (24.5–60.2)	0.4 (0.2–0.5)
	Hydrocoele	25 (8–42)	14.0 (3.32–24.7)	0.1 (0.03–0.2)
**Khulna**	All cases	17 (8–26)	6.1 (2.5–9.7)	0.07 (0.03–0.1)
**(n = 13)**	Lymphoedema	15 (7–24)	5.6 (2.1–9.1)	0.07 (0.02–0.1)
	Hydrocoele	0.9 (0.4–1.4)	0.3 (0.11–0.48)	0.003 (0.001–0.006)
**Rajshahi**	All cases	49 (20–78)	14.7 (8.5–21.0)	0.15 (0.1–0.2)
**(n = 36)**	Lymphoedema	37 (14–61)	11.1 (6.2–15.9)	0.12 (0.1–0.2)
	Hydrocoele	9.8 (4.0–16)	3.1 (1.4–4.8)	0.9 (0.6–1.1)
**Overall** **(n = 132)**	All cases	331 (233–429)	123.3 (89.6–157.1)	1.3 (0.9–1.6)
	Lymphoedema	232 (160–304)	84.5 (61.2–107.8)	0.9 (0.6–1.2)
	Hydrocoele	97 (66–128)	38.3 (25.6–50.9)	0.4 (0.2–0.5)

**Fig 2:**
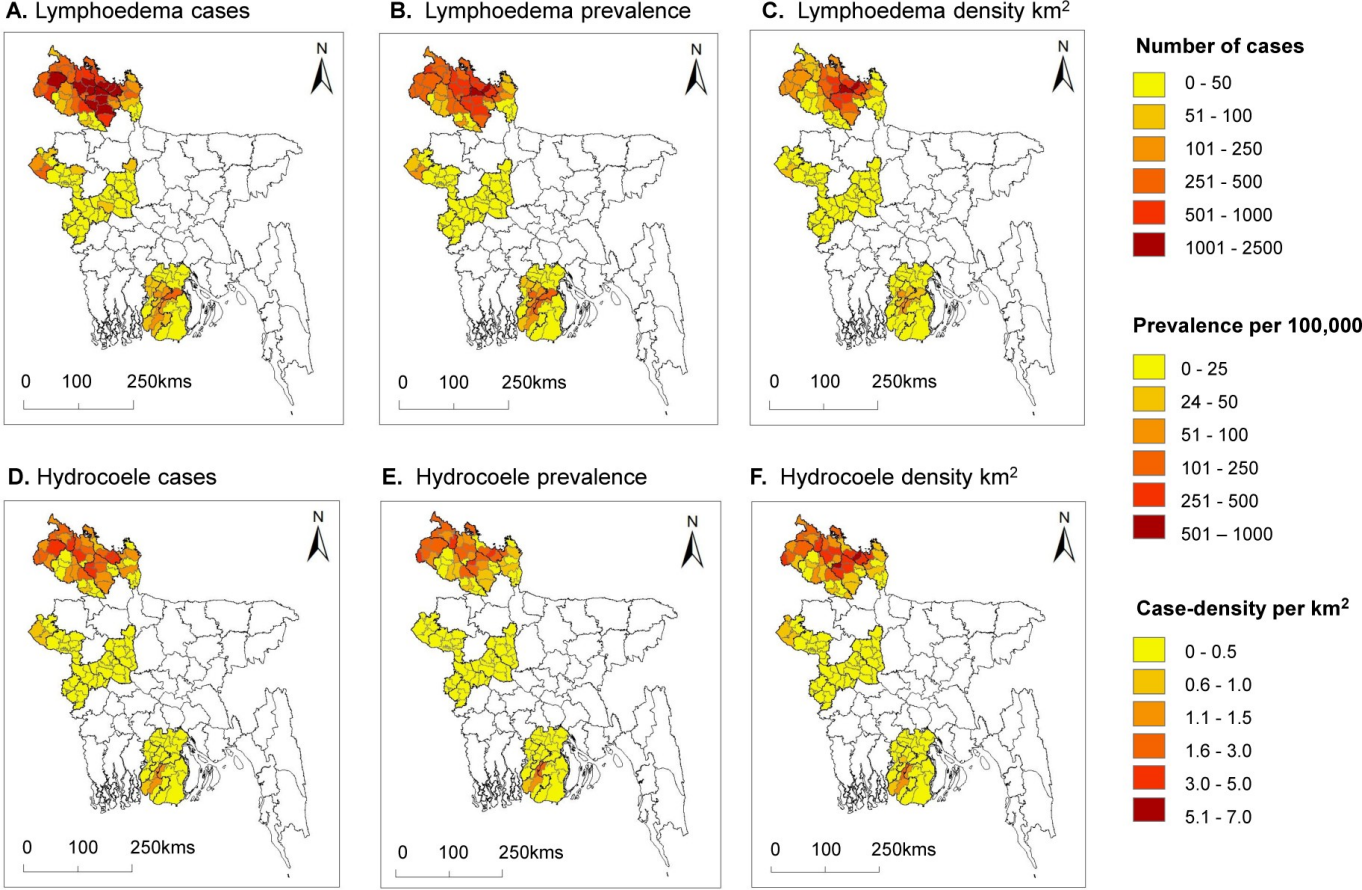
Clinical case numbers, clinical prevalence per 100,000 and case density per square kilometre (km^2^) by upazila. A. Lymphoedema case B. Lymphoedema prevalence C. Lymphoedema density km^2^ D. Hydrocoele cases E. Hydrocoele prevalence F. Hydrocoele density km^2^.

Distribution maps of upazila level lymphoedema and hydrocoele cases, clinical prevalence rates and case density rate are shown in Fig 2. For lymphoedema, the mean number of cases per upazila was 232 (95% CIs 160–304), prevalence rate per 100,000 was 84.5 (95% CIs 61.2–107.8) and 0.9 cases per km^2^ (95% CIs 0.6–1.2). The highest mean number, clinical prevalence and density rates were found in Rangpur Division, which were 525 (95% CIs 370–681), 182.8 (95% CIs 134.3–231.3) and 2.0 (95% CIs 1.3–2.5) respectively, and significantly higher than the other Divisions.

For hydrocoele, the overall mean number of cases was 97 (95% CIs 66–128), prevalence rate per 100,000 was 38.3 (95% CIs 25.6–50.9) and 0.4 cases per km^2^ (95% CIs 0.2–0.5) per upazila as shown in Table 6. The highest number of case numbers, clinical prevalence rates and case density rates were found in Rangpur Division, which were 229 (95% CIs 164–294), 87.9 (95% CIs 60.8–115.1) and 0.9 (95% CIs 0.6–1.1) respectively, and significantly higher than the other Divisions.

### Lymphoedema and hydrocoele hotspots

**Fig 3:**
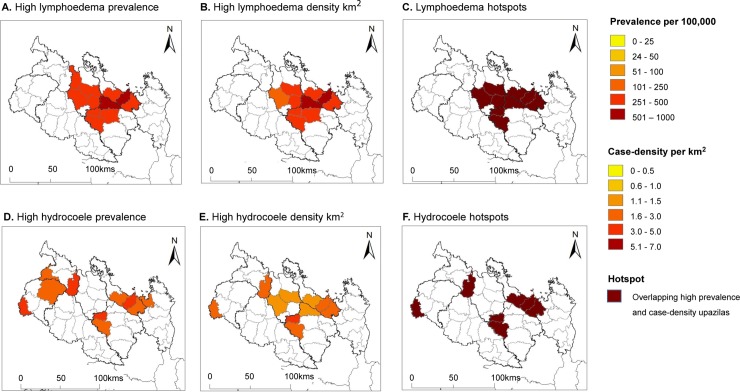
The highest clinical prevalence and case density per km2, and overlapping hotspots by upazila in Rangpur Division. A. High lymphoedema prevalence B. High lymphoedema density km^2^ C. Lymphoedema hotspots D. High hydrocoele prevalence E. High hydrocoele density km^2^ F. Hydrocoelehotspots.

The upazilas with the highest lymphoedema prevalence rates (range 121.7–752.7 per 100,000) and case-density rates (range 1.0–8.6 per km^2^) were all found in Rangpur Division (Fig 3). The highest rates were found to overlap in nine upazilas from three districts within one central geographical area defining a ‘lymphoedema hotspot’ and included Lalmonirhat District (Aditmari, Kaligang, Sadar upazilas), Nilphamari District (Jaldhaka, Kishoreganj, Sadar upazilas) and Rangpur District (Badarganj, Gangachara, Taraganj upazilas).

The upazila with the highest hydrocoele prevalence rates (range 129.3–486.3 per 100,000) and case-density rates (range 1.5–5.3 per km^2^) were all found in Rangpur Division (Fig 3). The highest rates were found to overlap in seven upazilas from four districts across the area to define four small geographically distinct ‘hydrocoele hotspots’, and included Lalmonirhat District (Aditmari, Kaligang, Sadar upazilas), Panchagarth District (Debiganj upazila), Rangpur District (Badarganj, Taraganj upazilas) and Thakurgaon District (Haripur upazila). The distinct geographical pattern of the hydrocoele hotspots may be explained by districts’ annual records on hydrocoele surgeries, which indicated in Rangpur Division (Dinajpur, Lalmonirhat, Nilphamari, Panchagar, Thakurgaon Districts) that 14,746 hydrocoele surgeries were performed in campaigns and at the filarial hospital between 2003 and 2012 (2003 n = 348; 2004 n = 288; 2005 n = 723; 2006 n = 436; 2007–2008; 10,003; 2009 n = 2248; 2010 n = 160; 2011 n = 300; 2012 n = 240). No specific details of location are available.

Five lymphoedema hotspots geographically overlapped with hydrocoele hotspots and included in Rangpur District the upazilas of Badarganj (lymphoedema 391.1 per 100,000 and 4.0 cases per km^2^; hydrocoele 233.0 and 2.4km^2^) and Taraganj (lymphoedema 410.0, 4.5 km^2^; hydrocoele 486.3, 5.4km^2^), and in Lalmonirhat District the upazilas of Kaligang (lymphoedema 426.7, 4.4 km^2^; hydrocoele 152.9, 1.6km^2^), Aditmari (lymphoedema 730.9, 8.5km^2^; hydrocoele 307.9, 3.6km^2^) and Sadar(lymphoedema 459.6, 5.8km^2^; hydrocoele 162.4, 2.1km^2^).

### Upazila-level age-specific and lymphoedema stage analysis

Reported data on patient’ age and lymphoedema stage were limited but available for the hotspot upazilas of Taraganj (Rangpur) and Haripur (Thakurgaon), which were examined together with two other (non-hotspot) upazilas with data available namely, Baliadangi (Thakurgaon) and Tatulia (Panchagarh). Cases with information on age and/or lymphoedema stage were included in the analysis.

In Taraganj, there were 539 leg lymphoedema and 701 hydrocoele cases, in Haripur 242 lymphoedema and 401 hydrocoele, in Baliadangi 332 lymphoedema and 219 hydrocoele, and Tatulia 76 lymphoedema and hydrocoele 146 cases. The number of lymphoedema cases was 2.0 to 4.6 times higher in females than males; Taraganj (96 male; 443 female), Haripur (65 male, 177 female), Baliadangi (111 male, 221 female) and Tatulia (27 male; 49 female).

**Fig 4:**
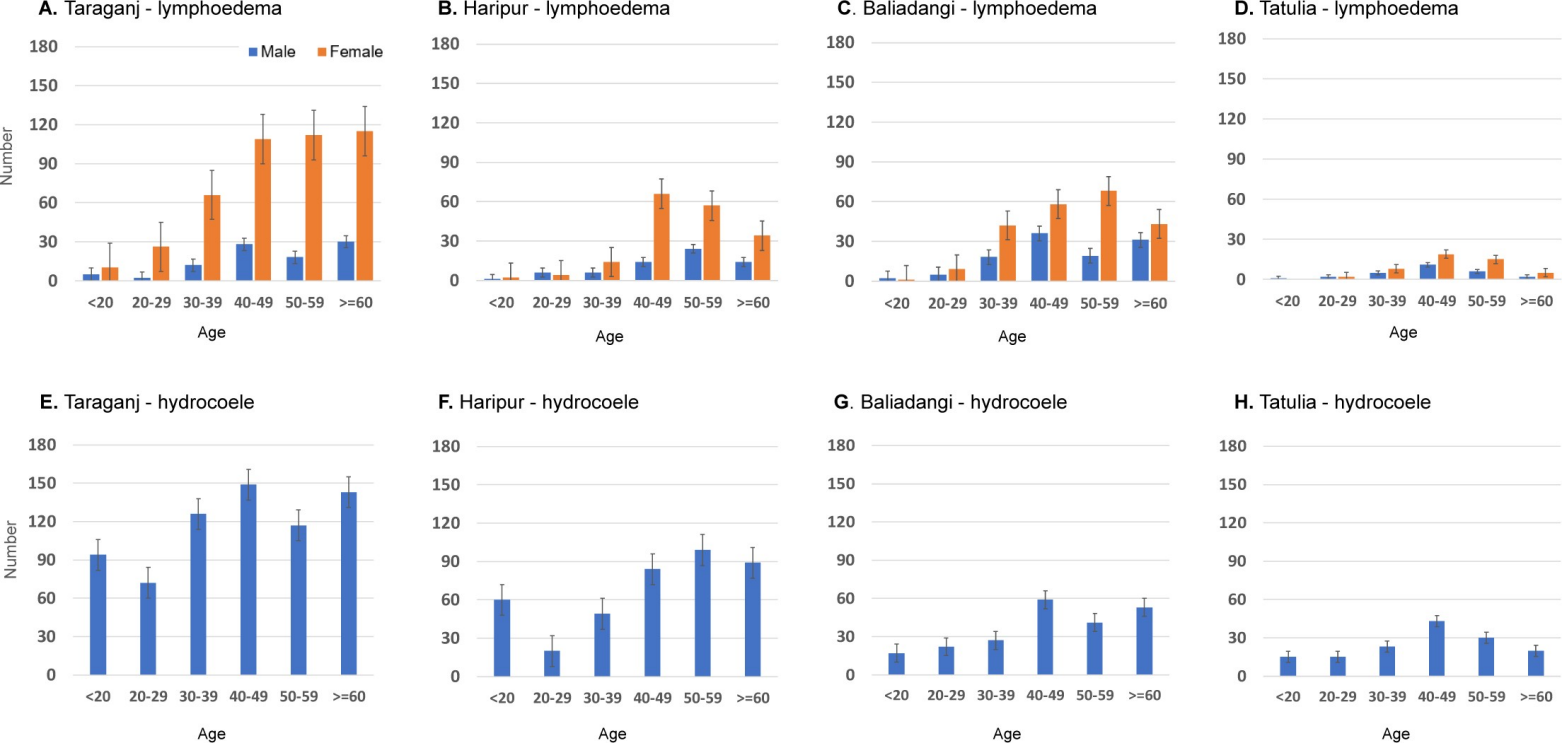
Distribution of lymphoedema and hydrocoele cases by upazila, sex, and age group. A. Taraganj–lymphoedema B. Haripur–lymphoedema C. Baliadangi–lymphoedema D. Tatulia—lymphoedema E. Taraganj–hydrocoele F. Haripur–hydrocoele G. Baliadangi–hydrocoele H. Tatulia–hydrocoele.

The average age of male and female lymphoedema cases, and hydrocoele cases were similar in all four upazilas; Taraganj lymphoedema (48.5 male, 47.9 female) and hydrocoele (41.5), Haripur lymphoedema (48.5 male, 48.9 female) and hydrocoele (43.2), Baliadangi lymphoedema (49.2 male, 48.7 female) and hydrocoele (46.0) and Tatulia lymphoedema (42.6 male; 48.2 female) and hydrocoele (41.3). The number of lymphoedema cases increased with age, especially among females as shown in Fig 4. The number of hydrocele cases also increased with age, however, the patterns were more variable, and possibility influenced by the number of surgeries conducted in the region as noted above.

**Fig 5:**
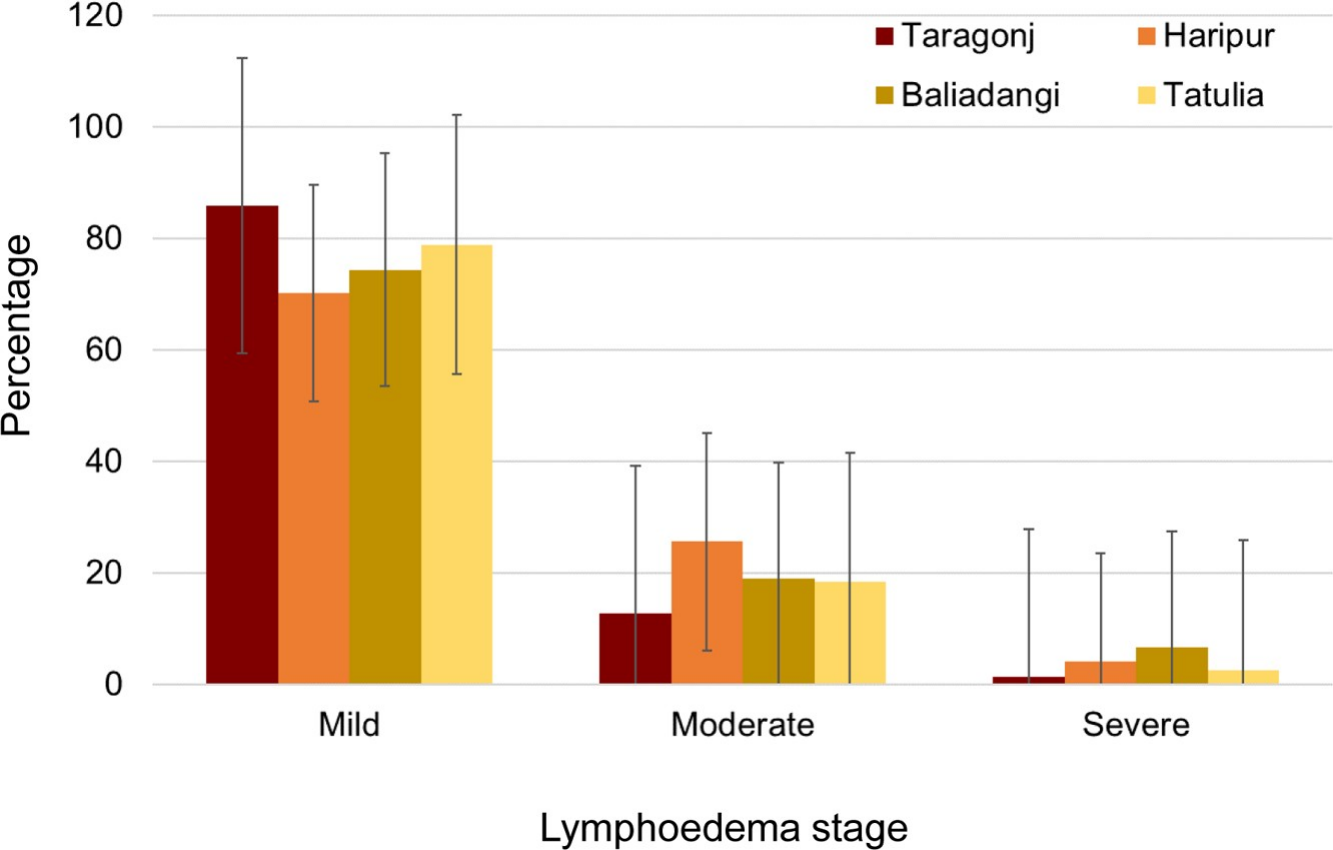
Proportion of mild, moderate and severe lymphoedema cases by upazila.

**Fig 6:**
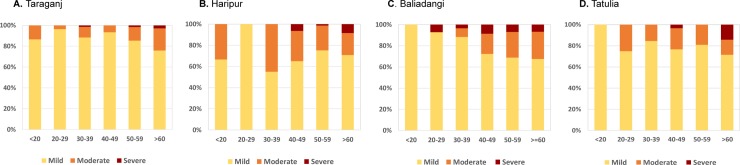
Proportion of mild, mild and severe lymphoedema cases by age group and upazila. A. Taraganj B. Haripur C. Baliadangi D. Tatulia.

Overall, the proportion of lymphoedema cases classified as mild, moderate and severe were similar across the four upazilas: Taraganj mild 85.9%; moderate 12.8%, severe 1.3%, Haripur 70.2%, 25.6%, 10 4.1%, Baliadangi 74.4%, 19.0%, 6.6% and Tatulia 78.9%, 18.4%, 2.6% (Fig 5). Overall, there was a higher proportion of moderate to severe cases in older age groups in all four upazilas; Taraganj 21.4% moderate and 2.8% severe in >60 age group), Haripur (20.8% moderate, 8.3% severe), Baliadangi (25.7% moderate, 6.8% severe) and Tatulia (14.3% moderate, 14.3% severe) as shown in Fig 6.

### Correlation between district baseline mf prevalence and clinical prevalence rates

Spearman’s correlations showed significant positive associations between the baseline mf prevalence and the overall prevalence 0.882 (p<0.01), lymphoedema prevalence 0.873 (p<0.01) and hydrocoele prevalence 0.858 (p<0.01). Linear regressions run to predict overall case prevalence, lymphoedema prevalence and hydrocoele prevalence resulted in adjusted R2 values of 0.848, 0.765 and 0.853 respectively.

## Discussion

This paper highlights the significant efforts of the Bangladesh LF Elimination Programme to train a large cadre of upazila and community clinic staff to identify and report people affected by LF in endemic areas. The extensive data collected has facilitated the development of the first national database and map of LF clinical cases in Bangladesh, which represents one of the most comprehensive databases available in the world. This provides an informative baseline database for the LF programme and local health workers to use with good estimates on the number of patients, which will help assess the readiness and quality of services to deliver the minimum package of care. It will also help to identify and target morbidity hotspots, which were defined based on a combination of prevalence and case density rates. While this comprehensive approach of patient searching required significant time and resources, it was considered better than other methods that potentially underestimate the real burden of disease [15,22,23]. It also provides a template for other countries to adopt and develop national strategies to manage morbidity and preventing disability as recommended by GPELF.

In the high endemic districts, the health worker training and patient searching activities were well planned and conducted in phases over a four-year period, covering an estimated 34.8 million people. The extensive training at the community clinics will facilitate the integration of LF clinical care into the primary health care system as they are expected to provide lymphoedema management at community level and encourage patients to access care and receive advise and counselling on their condition. The house-to-house patient census provides detailed information on the number and type of cases at the IU level, which is required for the validation dossier [13].

Importantly, the data provide a good foundation to develop post-elimination surveillance strategies to ensure the numbers progressively decline over time, and no new cases emerge. The programme is working with the Management Information System (MIS) department of the MOHFW to incorporate indicators into the District Health Information System (DHIS, version 2) to enable the long-term reporting of new cases, and patient access to care (lymphoedema management and hydrocele surgery) by health facilities. These indicators and reporting requirements will be incorporated into the next phase of health worker training to ensure proper use and data quality. The data will also help to ensure gender-sensitive patient care and treatment packages are implemented to address the different clinical conditions affecting males and female patients.

Most men with hydrocoele will need surgery, and the capacity of hospitals will need to be assessed to determine if they have the essential infrastructure, human and financial resources to conduct hundreds to thousands of operations over the next decade. A hospital facility assessment survey tool is currently being developed and will be trialled in endemic districts in 2019. This will help the LF programme identify the hospitals that are fit-for-purpose and able the scale-up surgeries, thus ensuring quality of care, as required by the GPELF [13]. The LF programme aims to significantly reduce the LF caused hydrocoele burden by 2030 as part of the updated national eradication plan, and it will be important that they target all age groups as we found young men also affected. To reduce the backlog of cases, the LF programme have conducted hydrocele surgery campaigns in high burden districts since 2016, delivering over 1200 surgeries to date, with another 1300 surgeries planned for 2019. This will be a significant achievement and improve the socio-economic status and quality of life of tens of thousands of men, their families and caregivers [24–28], especially in highly endemic districts such as Rangpur and Thakurgaon.

In contrast, the lymphoedema patients, who were predominately female, will need to implement home-based MMDP strategies for the rest of their lives in order to prevent ADLAs and the worsening of their condition [14,29]. Studies elsewhere have also shown that lymphoedema affects females more than males [30,31], however the reasons are not well understood and could be due to gender related exposure, sex hormones, or genetics [32]. More information on the impact of lymphoedema, why it affects women more, and how they can better manage the long-term disability is urgently required, as it has wide range of physical, social and economic affects [33]. Compared with people unaffected, lymphoedema patients have a significantly poorer quality of life, impeding their mobility, earning potential and ability to spend on necessary healthcare [34].

Overall, most patients were found to have mild stage of lymphoedema and will be able to manage their condition through home-based care. However, the smaller proportion of patients with severe stage lymphoedema, which largely affected older people, will require more specialised care, and may be more vulnerable to ADLAs as shown in other studies [35]. Information on ADLAs was not systematically collected and included in the current database, however clinic staff have since been advised to conduct lymphoedema management sessions regularly (fortnightly or monthly) to enable lymphoedema patients to access care, receive advice and counselling on their condition. Additionally, clinics are now equipped to provide treatment for ALDAs at any time.

The WHO recommends good hygiene, skin care, limb exercise and elevation, and proper footwear [11,12], which are effective low-tech interventions with improvements translating to socio-economic benefits and improved quality of life for patients, their families and caregivers [36–41]. However, as new alternative physical and therapeutic intervention strategies [42,43] become available and/or are recommended by the WHO, it will be important for the LF programme to adopt them and devise new plans to train health workers and reach patients, as they are likely to improve patients’ conditions more rapidly and for longer periods.

In the low endemic districts, the combination of mobile teams, community clinic staff and local informants found very low numbers of cases, which correlate with low baseline prevalences and recent negative TAS results [1]. This helps to confirm that LF is not a significant public health problem in these 15 districts, and a similar method could be used in the non-endemic districts if cases were suspected. Regardless, the community clinic health staff in these low endemic areas will still require training to implement patient care and treatment to ensure that no-one is left behind [44]. This may be challenging when cases are sparse, but the LF programme should aim to learn as much as possible from implementing MMDP activities in these low endemic communities, as they may reflect the future situation in highly endemic areas as case numbers progressively decline to zero. The use of the adapted patient searching method may be better use of human and financial resources [19].

Overall there was a significant correlation between mf and clinical prevalence rates, which suggests that mf prevalence data may be used to model and predict clinical burden in unmapped areas, with only selected field verification needed. This could help national programmes save time and resources in establishing the case estimates required for the dossier [13], and direct where detailed mapping may be required, and/or care and treatment packages needed most. National programmes should start in high sero-prevalence areas where the clinical burden is likely to be highest. However, it is important to note that the number of cases, and the relationship between mf and clinical prevalence, may vary across different epidemiological settings. Further, Bangladesh and other national programmes should aim to measure, monitor and model the decline of clinical prevalence and impact of interventions, similar to transmission models [45–47], as they will help predict the resources needed over time. The Bangladesh programme is planning an updated census and mapping of clinical cases every five years, especially in morbidity hotspot areas to help track progress and secure the government resources required to continue long-term essential care to patients.

This study found geographically well-defined morbidity hotspots in the northern Rangpur Division, which overlap with areas of high baseline prevalence and TAS positive children [1]. This high prevalence region is a high priority for the LF programme, and the collective use of the available sero-prevalence, TAS and morbidity datasets will help to develop targeted post-elimination surveillance strategies, which will need to be integrated into national health systems to ensure that the national and global elimination goals are reached and maintained over the next decade.

## Supporting information

S1 Checklist STROBE checklist. (DOCX)
