# Supplementary material for: Developing the first national database and map of lymphatic filariasis clinical cases in Bangladesh: Another step closer to the elimination goals
Source: PLoS Negl Trop Dis. 2019 Jul 15;13(7):e0007542. doi: 10.1371/journal.pntd.0007542 (PMC6658114; doi:10.1371/journal.pntd.0007542)
Supplement: S1 Checklist — (DOCX) [file pntd.0007542.s001.docx]

STROBE Statement—Checklist of items that should be included in reports of ***cross-sectional studies***

|  | Item No | Recommendation |
| --- | --- | --- |
| **Title and abstract** | 1 | (*a*) Indicate the study’s design with a commonly used term in the title or the abstract **Abstract** |
|  |  | (*b*) Provide in the abstract an informative and balanced summary of what was done and what was found **Abstract** |
| Introduction | | |
| Background/rationale | 2 | Explain the scientific background and rationale for the investigation being reported **Introduction, paragraphs 3,4** |
| Objectives | 3 | State specific objectives, including any prespecified hypotheses **Introduction, paragraphs 5,6** |
| Methods | | |
| Study design | 4 | Present key elements of study design early in the paper **Methods sections** - **Patient searching in high endemic districts and Patient searching in low endemic districts** |
| Setting | 5 | Describe the setting, locations, and relevant dates, including periods of recruitment, exposure, follow-up, and data collection **Methods sections - Survey sites, Health worker training, Patient searching in high endemic districts and Patient searching in low endemic districts** |
| Participants | 6 | (*a*) Give the eligibility criteria, and the sources and methods of selection of participants **Methods section** – **Health worker training, Patient searching in high endemic districts and Patient searching in low endemic districts** |
| Variables | 7 | Clearly define all outcomes, exposures, predictors, potential confounders, and effect modifiers. Give diagnostic criteria, if applicable **Methods section** - **Patient searching in high endemic districts and Patient searching in low endemic districts** |
| Data sources/ measurement | 8* | For each variable of interest, give sources of data and details of methods of assessment (measurement). Describe comparability of assessment methods if there is more than one group **Methods section** - **Patient searching in high endemic districts, Patient searching in low endemic districts, Data analysis, mapping and hotspot identification** |
| Bias | 9 | Describe any efforts to address potential sources of bias **Methods section** - **Patient searching in high endemic districts and Patient searching in low endemic districts** |
| Study size | 10 | Explain how the study size was arrived at **Methods section** - **Patient searching in high endemic districts and Patient searching in low endemic districts** |
| Quantitative variables | 11 | Explain how quantitative variables were handled in the analyses. If applicable, describe which groupings were chosen and why **Method sections – Data analysis, mapping and hotspot identification and Relationship between district baseline mf prevalence and clinical prevalence rates** |
| Statistical methods | 12 | (*a*) Describe all statistical methods, including those used to control for confounding **Method sections – Data analysis, mapping and hotspot identification and Relationship between district baseline mf prevalence and clinical prevalence rates** |
|  |  | (*b*) Describe any methods used to examine subgroups and interactions **Method section – Data analysis, mapping and hotspot identification** |
|  |  | (*c*) Explain how missing data were addressed **Not applicable** |
|  |  | (*d*) If applicable, describe analytical methods taking account of sampling strategy **Not applicable** |
|  |  | (*e*) Describe any sensitivity analyses **Not applicable** |
| Results | | |
| Participants | 13* | (a) Report numbers of individuals at each stage of study—eg numbers potentially eligible, examined for eligibility, confirmed eligible, included in the study, completing follow-up, and analysed **Results sections – District-level training, case numbers; Prevalence rates and Upazila-level case numbers, prevalence rates, case-density rates; Tables 1-3** |
|  |  | (b) Give reasons for non-participation at each stage **Not applicable** |
|  |  | (c) Consider use of a flow diagram **Not applicable** |
| Descriptive data | 14* | (a) Give characteristics of study participants (eg demographic, clinical, social) and information on exposures and potential confounders **Results sections – District-level training, case numbers; Prevalence rates and Upazila-level case numbers, prevalence rates, case-density rates; Tables 1-3** |
|  |  | (b) Indicate number of participants with missing data for each variable of interest **Not applicable** |
| Outcome data | 15* | Report numbers of outcome events or summary measures **Results section – District-level training, case numbers and prevalence rates; Upazila-level case numbers, prevalence rates, case-density rates; Tables 1-4** |
| Main results | 16 | (*a*) Give unadjusted estimates and, if applicable, confounder-adjusted estimates and their precision (eg, 95% confidence interval). Make clear which confounders were adjusted for and why they were included **Results sections – District-level training, case numbers and prevalence rates; Upazila-level case numbers, prevalence rates, case-density rates; Table 4** |
|  |  | (*b*) Report category boundaries when continuous variables were categorized **Results sections – District-level training, case numbers and prevalence rates; Upazila-level case numbers, prevalence rates, case-density rates; Table 4; Figures 2-3** |
|  |  | (*c*) If relevant, consider translating estimates of relative risk into absolute risk for a meaningful time period **Not applicable** |
| Other analyses | 17 | Report other analyses done—eg analyses of subgroups and interactions, and sensitivity analyses **Not applicable** |
| Discussion | | |
| Key results | 18 | Summarise key results with reference to study objectives **Discussion, paragraph 1** |
| Limitations | 19 | Discuss limitations of the study, taking into account sources of potential bias or imprecision. Discuss both direction and magnitude of any potential bias **Discussion, paragraphs 1,2,5** |
| Interpretation | 20 | Give a cautious overall interpretation of results considering objectives, limitations, multiplicity of analyses, results from similar studies, and other relevant evidence **Discussion, paragraphs** |
| Generalisability | 21 | Discuss the generalisability (external validity) of the study results **Discussion, paragraph, 2-6** |
| Other information | | |
| Funding | 22 | Give the source of funding and the role of the funders for the present study and, if applicable, for the original study on which the present article is based **Included in the** **funding information section on online submission** |

*Give information separately for exposed and unexposed groups.

**Note:** An Explanation and Elaboration article discusses each checklist item and gives methodological background and published examples of transparent reporting. The STROBE checklist is best used in conjunction with this article (freely available on the Web sites of PLoS Medicine at http://www.plosmedicine.org/, Annals of Internal Medicine at http://www.annals.org/, and Epidemiology at http://www.epidem.com/). Information on the STROBE Initiative is available at www.strobe-statement.org.
